# Supplementary material for: Ferroelectricity promoted by cation/anion divacancies in SrMnO3
Source: J Mater Chem C Mater. 2021 Sep 2;9(38):13321–30. doi: 10.1039/d1tc02317a (PMC8495715; doi:10.1039/d1tc02317a)
Supplement: TC-009-D1TC02317A-s001 [file TC-009-D1TC02317A-s001.pdf]

Electronic Supplementary Material (ESI) for Journal of Materials Chemistry C.
This journal is © The Royal Society of Chemistry 2021

Supplementary information for

Ferroelectricity promoted by cation/anion divacancies in $SrMnO_3$

Chiara Ricca,[1,2], Danielle Berkowitz[1] and Ulrich Aschauer[1,2]
[1] *Department of Chemistry and Biochemistry, University of Bern, Freiestrasse 3, CH-3012 Bern, Switzerland*
[2] *National Centre for Computational Design and Discovery of Novel Materials (MARVEL), Switzerland*

(Dated: May 19, 2021)

S1. STRAIN-DEPENDENCE OF SMO BAND-GAP

For the metallic FM phase, compressive strain results in a small band gap (E_g) almost linearly increasing with increasing strain (see Fig. S1). Instead, for the AFM phase, E_g increases up to -4% and decreases for larger strains, which strongly destabilizes this magnetic order.

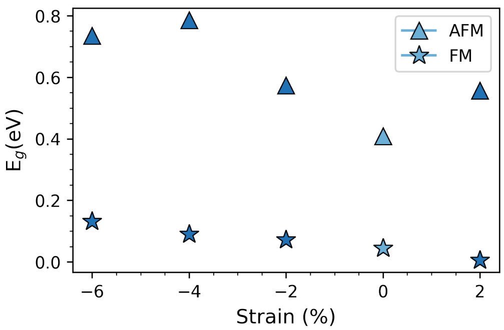

Figure S1. Evolution of the SMO bandgap (E_g) in the AFM and FM phase as a function of biaxial strain.

S2. ELECTRONIC PROPERTIES FOR $V_{SR}-V_O$ DEFECT PAIRS IN SMO

Due to the formal charge compensation of the V''_{Sr} and $V_O^{\bullet\bullet}$ defects, the formation of a neutral $V_{Sr}-V_O$ defect pair should not be associated with any excess charge (resulting, for example, in the reduction of Mn sites). However, the density of states reported in Fig. S2 for a $V_{Sr}-V_O^{IP}$ and a $V_{Sr}-V_O^{OP}$ suggests a partial reduction of one and two, respectively, Mn atoms adjacent to V_O forming $Mn^{(3+\delta)+}$ ions as a consequence of the structural relaxations taking place upon V_O formation. In particular, in the case of V_O^{OP}, after breaking the two equivalent Mn−O−Mn bonds along the *b*-axis, the remaining Mn−O bonds along this direction for the Mn in nearest neighbor position to the O vacancy are elongated by about 0.10 Å and reach 2.01 Å. This lowers the energy of the e_g states of these sites, which lie at the bottom of SMO conduction band. The lowering of the energy of these e_g orbitals to just below the Fermi energy results in partial filling of these states and consequently in a partial Mn reduction. This, however, is a consequence of the underestimation of the SMO band gap in DFT+U calculations[1]. Similar arguments apply to $V_{Sr}-V_O^{IP}$, but in this case only one of the two Mn ions adjacent to the removed O atom has the remaining Mn−O bond elongated by about 0.05 Å to reach 2.01 Å along the Mn−V_O−Mn axis. For the other site adjacent to the oxygen vacancy the remaining Mn−O bond is shortened by 0.13 Å to a relaxed length of 1.79 Å. As a result, only one Mn is artificially reduced.

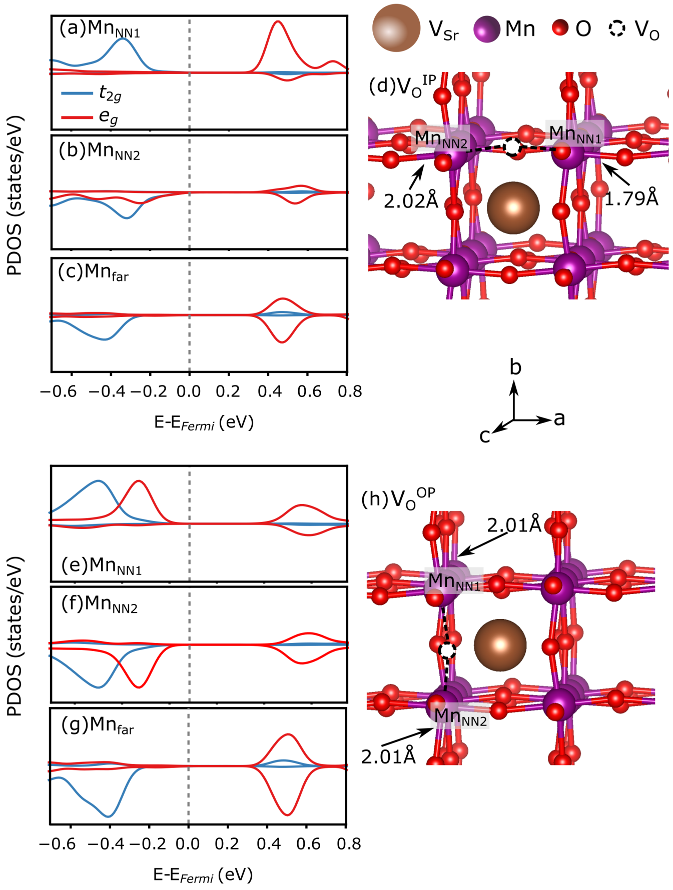

Figure S2. Atom- and orbital-projected density of states (PDOS) for the Mn-3d states of the Mn sites in nearest-neighbor (NN) positions to V_O (a,b,e,f) and for a Mn atom far from the defect (c and g) for a $V_{Sr}-V_O^{IP}$ (a-c) or a $V_{Sr}-V_O^{OP}$ (e-g) defect pair. The vertical dashed line indicates the Fermi level. Panels d) and h) shows the most relevant Mn−O bond changes taking place on the NN Mn atoms after $V_{Sr}-V_O^{IP}$ and $V_{Sr}-V_O^{OP}$ formation, respectively.

S3. STRUCTURAL RELAXATIONS AND MN OFF-CENTERINGS

Structural relaxations upon formation of a $V_{Sr}-V_O$ defect pair in the unstrained SMO structure mainly result in off-centering of Mn atoms in nearest neighbor (NN) positions to V_{Sr} from their high symmetry positions and towards the cation vacancy (see Figs. S3a and c). When V_{Sr} is NN to V_O, the Mn adjacent to both vacancies cannot move towards V_{Sr} because of the broken Mn−O−Mn bond. Overall the defect polarizes the surrounding atoms resulting in a polarization vector mainly aligned with the defect dipole, but forming a small angle with it as a consequence of the displacements of the Mn atoms in next nearest neighbor (NNN) positions to V_O along the axis where the Mn−O−Mn bonds is broken. Instead, for configurations where V_{Sr} lies at distances larger than 6 Å from V_O, a full contraction of the lattice, due to the displacements of all the Mn sites adjacent to V_{Sr} towards the vacancy, results in the polarization not being aligned with the defect dipole. For a larger tensile strain of 4%, when the in-plane polar modes are unstable, larger off-centering for all the Mn atoms are observed along the [101] direction: this effect partially enhances or counteracts the relaxations of Mn adjacent to V_{Sr} when the displacement due to the cation vacancy formation is aligned or opposite to the in-plane polarization direction (see Figs. S3b and d).

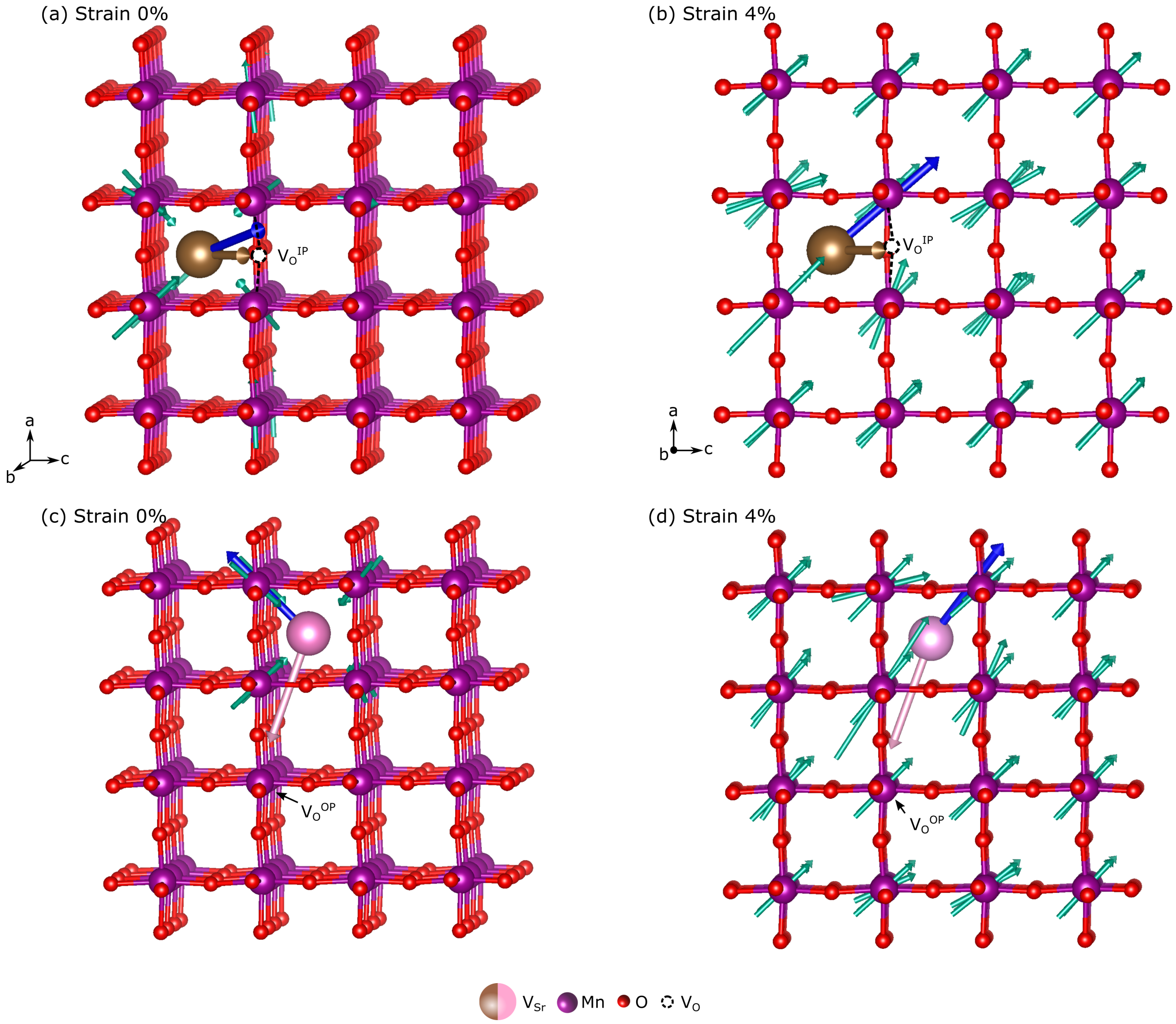

Figure S3. Off-centerings of the Mn atoms upon formation of a-b) one $V_{Sr}-V_O^{IP}$ and c-d) one $V_{Sr}-V_O^{OP}$ in unstrained SMO (plots a) and c) and for 4% tensile strain (plots b) and d). The green arrows indicate the vector corresponding to the off-centering of each Mn atom, while the brown/pink and blue arrows correspond to the defect dipole and polarization vectors, respectively.

S4. POLARIZATION IN THE FM ORDER

The strain dependent polarization of SMO with $V_{Sr}-V_O$ defect pairs is the result of a complex interplay between defect chemistry and electronic and magnetic degrees of freedom. While for the insulating AFM phase, polarization can be enhanced by tensile strain, in the metallic FM phase the polarization is fairly constant with respect to the applied epitaxial strain in the range between 0 and 6% (see Fig. S4). Interestingly, compressive strain beyond -2% can result in an enhancement of the out-of-plane component of the polarization, in line with the larger instability of the OP polar modes observed already in the stoichiometric FM phase (cf. Fig. 2 in the main text). In summary, the behavior of the FM phase can be rationalized considering both the larger electronic screening of the defect dipole in the metallic FM order, which results in a lower sensitivity of the polarization of the defective cells to the applied strain, and the strain-dependence of the polar modes frequencies in the stoichiometric FM SMO.

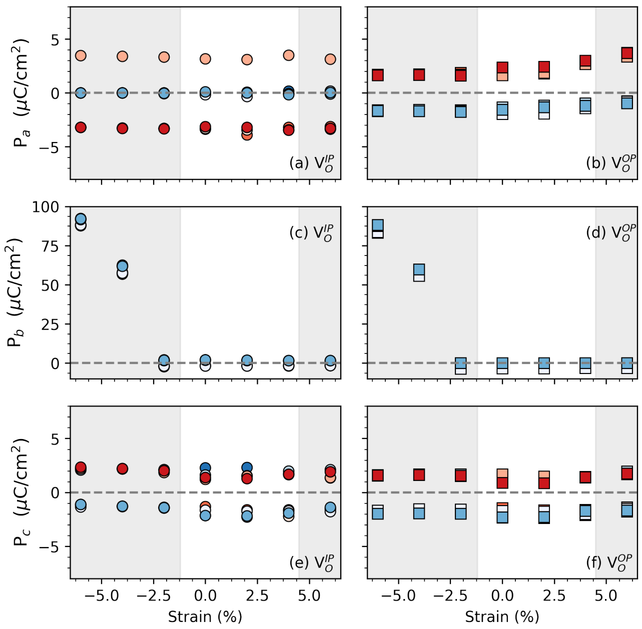

Figure S4. Strain dependence of the component of the polarization along the (a-b) *a*-, (c-d) *b*-, and (e-f) *c*-axis for the different defect-pair configurations in the FM phase of SMO. (a), (c), and (e) plots for $V_{Sr}-V_O^{IP}$ and (b), (d), and (f) plots for $V_{Sr}-V_O^{OP}$ defects. See Fig. 3 in the main text for the color code. The shaded grey areas indicate strain ranges with unstable polar modes in stoichiometric SMO.

[1] C. Ricca, I. Timrov, M. Cococcioni, N. Marzari, and U. Aschauer, Phys. Rev. B **99**, 094102 (2019).
